# Aptamer-Based Nanoporous Anodic Alumina Interferometric Biosensor for Real-Time Thrombin Detection

**DOI:** 10.3390/s19204543

**Published:** 2019-10-19

**Authors:** Laura Pol, Laura Karen Acosta, Josep Ferré-Borrull, Lluis F. Marsal

**Affiliations:** Departament d’Enginyeria Electrònica, Elèctrica i Automàtica, ETSE, Universitat Rovira i Virgili, Avda. Països Catalans 26, 43007 Tarragona, Spain; laura.pol@urv.cat (L.P.); laurakaren.acosta@urv.cat (L.K.A.); josep.ferre@urv.cat (J.F.-B.)

**Keywords:** nanoporous anodic alumina, streptavidin, aptamers, thrombin, RIfS, biosensing

## Abstract

Aptamer biosensors are one of the most powerful techniques in biosensing. Achieving the best platform to use in aptamer biosensors typically includes crucial chemical modifications that enable aptamer immobilization on the surface in the most efficient manner. These chemical modifications must be well defined. In this work we propose nanoporous anodic alumina (NAA) chemically modified with streptavidin as a platform for aptamer immobilization. The immobilization of biotinylated thrombin binding aptamer (TBA) was monitored in real time by means of reflective interferometric spectroscopy (RIfS). The study has permitted to characterize in real time the path to immobilize TBA on the inner pore walls of NAA. Furthermore, this study provides an accurate label-free method to detect thrombin in real-time with high affinity and specificity.

## 1. Introduction

To address the need of achieving rapid, reliable and low-cost systems for diagnosis or bio-detection in biomedical investigations, over the last few decades several methods for diagnosis, food safety or environmental monitoring have been investigated [1]. Biosensor devices have been reported as fast, selective and sensitive methods in bio-detection applications [2]. A biosensor consists of a device which converts a biological signal in an assessable response. The main components of biosensors are a bioreceptor, a transducer and a signal processor [3].

Depending on the transduction method, transducers can be classified in optical, mass-based or electrochemical biosensors and within these three categories, biosensors can be further classified as labelled or non-labelled biosensors [4]. On the other hand, the bioreceptors can be classified in several major categories depending on the reception molecules (e.g., antibodies, enzymes, cells, DNA, biomimetics and phages). Antibodies and DNA bioreceptors have been widely used in the last years in biosensor applications providing high sensitivity and specificity [5,6,7], while aptamers have been reported as advantageous over antibodies. Aptamers are single-strand DNA or RNA molecules with high specificity and affinity to their targets [8]. It has been proved that aptamers are able to bind with a broad range of analytes (e.g., cells, proteins, organelles, toxins, small molecules...) [8,9,10,11,12,13]. They are selected in-vitro by the Systematic Evolution of Ligands by Exponential Enrichment (SELEX) technique [14]. It provides the selection of the DNA or RNA molecule that will bind specifically with the target object of study, with dissociation constants between micromolar and picomolar range [15]. Since aptamers are synthetized in-vitro, it is not necessary to use animals for their production, they do not suffer from batch-to-batch variations and can be chemically modified on demand. Furthermore, although aptamers can be denaturalized as it happens with antibodies, for the case of aptamers such a process is reversible. Other advantages are their stability under a wide range of physiological and non-physiological conditions or, due to their small size, their ability to access binding sites not available to antibodies [8].

Biosensors engineered with aptamers as a bioreceptor are called aptabiosensors, they can be modified for immobilization purposes and can incorporate particular reporters, without influencing their affinity [2,16,17]. In addition, they can be easily labelled for their use in diagnostics [18]. Thrombin Binding Aptamer (TBA), whose DNA sequence is 5′GGT TGG TGT GGT TGG-3′, was selected by the SELEX technique by Bock and co-workers in 1992 [19]. Because of its well-known binding process and its high affinity, TBA is one of the most studied aptamers in biosensor probes [11,20,21]. Thrombin is the key factor of blood coagulation, whose activity is important in wounds and in blood coagulation disorders (e.g., hemophilia, coronary artery disease, diabetes or hyperprothrombinemia) [22]. Detecting the thrombin level will help to judge the coagulation ability of blood and prevent diseases such as thrombus. The biorecognition event works in the same manner as other aptamer biosensors, free thombin-binding aptamer remain as a random-coil state in the absence of thrombin, upon binding to thrombin, the conformation of TBA varies from random coil to quadruplex state, which is specifically binding with thrombin [13].

Regarding to the transducer methods, optical biosensors are biosensors based on the interaction of light with the biorecognition element. Within the field of optical biosensors, several optical label-free biosensors based on aptamers are reported in the literature. For instance, surface plasmon resonance (SPR) [9,23], localized surface plasmon resonance (LSPR) [24], ellipsometry [6], Surface-enhanced Raman spectroscopy SERS [18]. The reflection interference spectroscopy (RIfS) technique has demonstrated its capability to monitor biomolecule interactions in real time in porous silicon [25,26,27,28] and in nanoporous anodic alumina (NAA) with different kinds of targets [29,30,31].

Nanoporous anodic alumina (NAA) is a material of growing interest for use as a biosensor platform [32,33,34]. NAA is a self-ordered porous material with parallel pores growing perpendicularly to the surface [35]. NAA is a cost-effective material with well-defined and controllable geometry. Furthermore, NAA is an easily tunable material which provides a biocompatible platform with high surface-to-volume ratio, high chemical resistance, thermal stability and hardness [36]. Moreover, NAA possesses unique optical and electrochemical properties [37].

Because of its surface chemical properties, NAA can be chemically modified by the introduction of foreign functional groups able to further bind covalently or not-covalently with other biomolecules. For instance, the introduction of carboxyl (-COOH) or amino (-NH_2_) groups, together with the silanization process with aminopropyltriethoxy silane (APTES) [38,39,40], mercaptopropyl-triethoxy silane [36], polyethylene glycol-silane (PEG-silane) [34,36] and several other silanes have been studied.

Avidin-biotin interaction is one of the strongest non-covalent interactions in the nature. Streptavidin is an analogue of avidin that is extracted from *Streptomyces avidinii* that is a tetrameric protein able to bind with four biotins with a dissociation constant of K_d_ = 10^−14^ M. Furthermore, the streptavidin-biotin complex can be stable for 35 h [41]. Biotin is also a very stable molecule and easy to stick to most of biomolecules, and a wide range of commercial biotinylated molecules for biosensor applications is available.

One of the strategies to immobilize the TBA in the surface of a bioreceptor is through the biotin-streptavidin complex. Biotinylated TBA studies immobilized on several biosensor surfaces have been reported [12,21]. Because of the properties described above, NAA with streptavidin as a crosslinker in the surface provides a very useful platform to immobilize biotinylated molecules, particularly biotinylated aptamers [42].

In this work we first study the TBA immobilization into the inner surface of NAA pores through streptavidin-biotin interaction using the 15-mer-TBA sequence modified with biotin in position 5′ (5′-biotin-GGT TGG TGT GGT TGG-3′) by a three-stage process: first sulfo-NHS-biotin is grafted to the -NH_2_ of APTES, second streptavidin is attached to this sulfo-NHS-biotin on the NAA surface and third biotinylated TBA binds to the available sites of the surface-immobilized streptavidin. We study such immobilization stages with the RIfS technique to evaluate both the capability of such technique to sense such binding events and to quantify them. We then also evaluate the capability of the RIfS technique in a sensing stage to detect and quantify thrombin after the TBA immobilization. 

## 2. Materials and Methods

### 2.1. Materials 

Aluminum foils of 99.999% of purity and 0.5 mm of thickness were purchased from Goodfellow Ltd. (Cambridge, UK). Oxalic acid (H_2_C_2_O_4_), phosphoric acid (H_3_PO_4_), perchloric acid (HClO_4_), chromic acid (H_2_CrO_7_), copper chloride (CuCl_2_) ethanol (C_2_H_5_OH), acetone ((CH_3_)_2_CO), 2-(N-morpholino)ethanesulfonic acid, phosphate buffered saline (PBS), human serum albumin (HSA), streptavidin, (3-aminopropyl)triethoxysilane (APTES), sulfo-NHS-biotin, magnesium chloride (MgCl_2_) and thrombin were purchased from Sigma-Aldrich (St. Louis, MO, USA). Biotin modified aptamer 15-mer (5′biotin-GGT TGG TGT GGT TGG-3′) was purchased from Eurofins Genomic GmbH (Ebersberg, Germany). Double-deionized (DI) water Double-deionized (DI) water in 18.6 MU, PURELAB Option-Q system purchased from ELGA LabWater (Lane End, United Kindom) was used for all solutions.

### 2.2. NAA Preparation

NAA samples were prepared by anodization of aluminum foils following the well-known two-step anodization method with 0.3 M of oxalic acid at 40 V and 5 °C previously described in the literature [43,44]. Anodization was carried out with a SourceMeter model 2400 from Keithley Instruments Inc. (Cleveland, OH, USA) using the aluminium foils as anode and a platinum wire as cathode. The SourceMeter fixed the potential difference between anode and cathode at the mentioned 40 V while providing and measuring the required current for anodization. Aluminum foils of 99.999% purity and 0.5 mm thickness purchased from Goodfellow Cambridge Ltd were used. In the first step a first alumina layer was formed by anodization for 20 h, and subsequently this alumina layer was removed in etching solution of H_3_PO_4_ 6% wt and H_2_CrO_7_ 1.8% wt at 70 °C for 3 h. The resulting aluminium foil shows a surface patterned with concavities at the sites the pores have growth in the first step. This texturized aluminium foil was used as the anode in a second anodization step carried out at the same bias conditions as the first step. The process was applied until a total charge of Q= 20 C circulated through the electrochemical system. This resulted in a NAA pores with approximately 5 µm depth and 30 nm pore diameter. The pore diameter was adjusted to 60 nm by immersion of NAA in 0.3 M H_3_PO_4_ at 35 °C for 20 min. Samples were inspected by ESEM to assess the uniformity of pore sizes and lengths (Supporting Information, Figure S1)

### 2.3. Amino-NAA Surface Preparation

**Figure 1:**
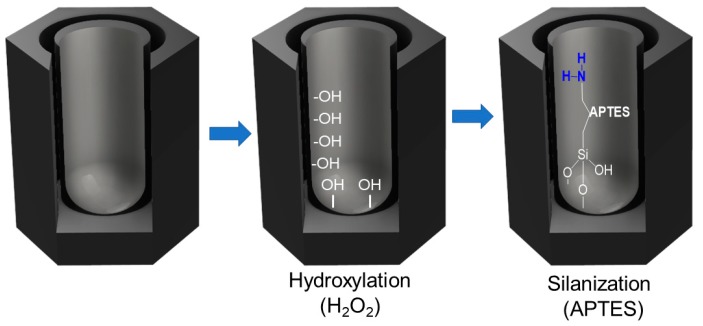
Schematics of the immobilization of APTES into the pores of NAA.

To use as a functional amino-crosslinking surface, the NAA samples were chemically pre-treated with APTES as is illustrated in Figure 1 and following the reported experimental procedure [38,39,40].

To do that, firstly NAA were hydroxylated by immersion in boiling hydrogen peroxide (H_2_O_2_) for 1 h with continuous stirring. Then the samples were washed with ethanol and water and dried by blowing with nitrogen. The NAA was then incubated under continuous stirring for 1 h in 15 mL of anhydrous toluene and 1.5 mL of APTES. Then NAA was sonicated in toluene for 5 min to remove non-linked APTES. The NAA with APTES was washed with ethanol and dried with nitrogen and placed in oven overnight at 110 °C. Subsequently, a thin film of gold with 10 nm thickness was deposited by sputtering on the NAA upper surface. This film of gold permits to increase the refractive index contrast between the NAA and the bound molecules to increase the sensitivity of the system without affecting the inner pore surface properties of NAA [40,45].

### 2.4. Reflectometric Interference Spectroscopy System (RIfS)

**Figure 2:**
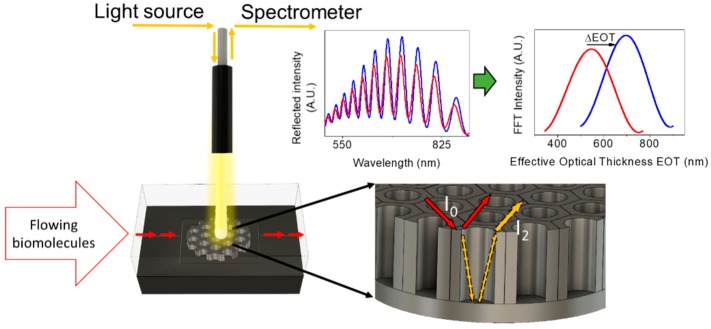
Explicative scheme of the Reflectometric Interference Spectroscopy system (RIfS).

APTES-NAA (NAA-HN_2_) foils were placed in a transparent flow cell system based on RIfS. In this system the fluid with the dissolved analytes are injected in the cell and put in contact with the NAA, allowing the analyte to diffuse into the inner NAA pores and interact with the amino-activated surface. Simultaneously, light is directed to the NAA surface and propagates within the NAA porous layer filled with the fluid. The NAA have two different interfaces one on top and one on the bottom of the pores when the light arrives to NAA, part of it is reflected at the top interface while another part travels across the pore and then is reflected at the bottom interface. When these two reflected beams overlap at the detector, Fabry-Pérot-like interferences arise because of their optical path difference, which depends of the light wavelength and the effective refractive index of the porous film. The reflected light is collected by a spectrometer which generates an interferometric reflectance spectrum which can be analysed by a Fourier transform that permits to extract the effective optical thickness (EOT) of the porous film. Figure 2 shows an explicative scheme of this technique.

### 2.5. Real Time Monitoring of Streptavidin-Mediated Biotinylated Aptamer Immobilization into NAA Pores

**Figure 3:**
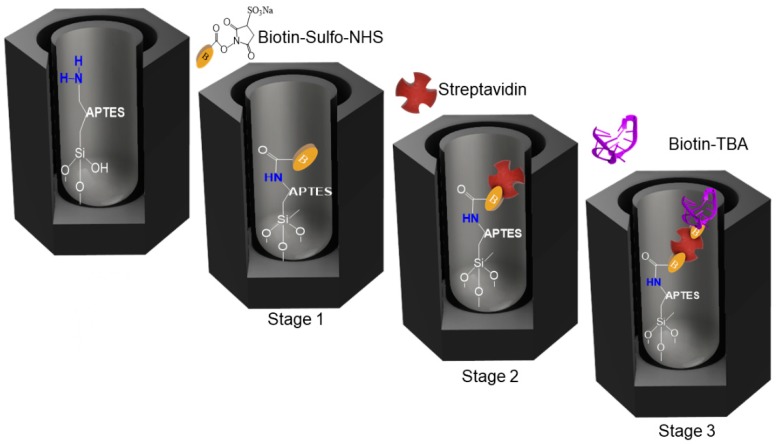
Schematics of aptamer immobilization into NAA pores.

APTES-NAA were placed in the flow cell to monitor in real time the attachment of thrombin binding aptamer (TBA) to the inner pore walls through a three-stage biotin-streptavidin interaction, illustrated in Figure 3.

In the first stage, the free amino groups of APTES were coupled to sulfo-NHS-biotin by the formation of an amide bond. To do that, 500 µl of 5 mM sulfo-NHS-biotin diluted in PBS was injected to the flow cell and recirculated for 30 min, followed by washing step with PBS. The second stage consisted in flowing 1 mL of a 50 µg/mL streptavidin solution and recirculating it for 60 min, followed by a washing step with binding buffer (PBS+MgCl_2_). A different buffer is used in this second stage as well as in the third stage because MgCl_2_ is used to stabilize the DNA. Finally, the third stage consists of the flow of 500 µL of biotinylated TBA diluted in binding buffer to 10 mM. Previously to the introduction into the flow cell, the biotinylated TBA was thermally treated by incubation in binding buffer for 5 min at 95 °C and cooled at room temperature for 15 minutes adapting the protocol from literature [46,47,48]. After this three-stage functionalization process, Aptamer-NAA foils were exposed to the flow of thrombin solutions at different concentrations.

## 3. Results and Discussion

### 3.1. Study of NAA Surface Functionalization with Biotin-Modified TBA Aptamer

**Figure 4:**
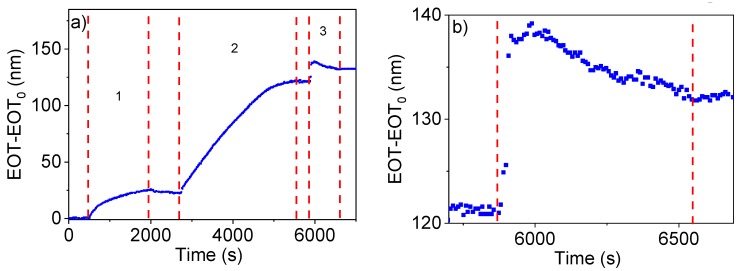
(**a**) Registered change in EOT as a function of time during NAA surface functionalization: (1) sulfo-NHS-biotin, (2) streptavidin, (3) trombin-binding-aptamer (TBA). (**b**) A close-up of the EOT variation with time of the step 3 of the experiment in Figure 4a.

Figure 4a shows the change in EOT as a function of time during the surface functionalization of NAA by the covalent attachment of sulfo-NHS-biotin and subsequent streptavidin attachment (stages 1 and 2 in the figure) and the final immobilization of TBA in the pore walls of NAA (step 3 in the plot).

When the sulfo-NHS-biotin solution was flowed in the first stage, a considerable increase in EOT of about 25 nm was observed until a stable value was achieved after approximately 1800 s. The washing step with PBS in this first stage produced a very small decrease in EOT. With the infiltration of streptavidin in the second stage, a rapid increase in EOT of about 100 nm in 3600 s was produced. After this increase, the EOT remained stable for 230 s. Finally, when the biotinylated TBA solution was flowed in the third stage, a marked increase of about 15 nm of EOT is produced in 120 s. After this initial increase, the EOT decreases and stabilizes with a total increase of 15 nm in this stage. After the aptamer immobilization, a final washing with binding buffer was performed to increase the stability of DNA and eliminate the non-bounded TBA molecules. 

This procedure was applied in the same conditions to all the samples used in this work, as they were intended for the detection of different concentrations of thrombin. It was observed that the evolution of EOT in the three steps was highly reproducible among experiments (experiments showed in Supporting Information, Figure S2). 

In the first stage of Figure 4a it was observed that the introduction of sulfo-NHS-biotin produces a marked increase in EOT which soon stabilizes due to the covalent coupling of all available -NH_2_ groups of APTES with the -NHS group of the biotin forming an amide bond. When in the stage 2 streptavidin was introduced and bound whit biotin, we observed that the stabilization of EOT takes twice as long as in stage 1 and it results in a higher increase in EOT and with a higher increase rate. The differences in behaviour between the two stages can be due to two reasons: (i) the different type of coupling: in stage 1 a covalent bond takes place, resulting a shorter reaction time whereas in stage 2, the biotin-streptavidin interaction is slower, and (ii) streptavidin has a much bigger size than biotin, resulting in a higher increment of EOT and with a bigger slope.

In stage 3 when the aptamer was flowed, an initial rapid increase in EOT was produced followed by a small decrease after a few s and stabilizing at an EOT sensibly above the value at the end of the previous stage. This behaviour can be explained in terms of the ability of DNA molecules to change their conformation: the initial steep increase in EOT can be related to the binding event, while the subsequent slow decrease may indicate that after binding the molecules change their conformation, leading to a decrease in their effective refractive index.

### 3.2. Detection of Thrombin with Aptamer-Functionalized NAA. Study of Linearity and Sensitivity

**Figure 5:**
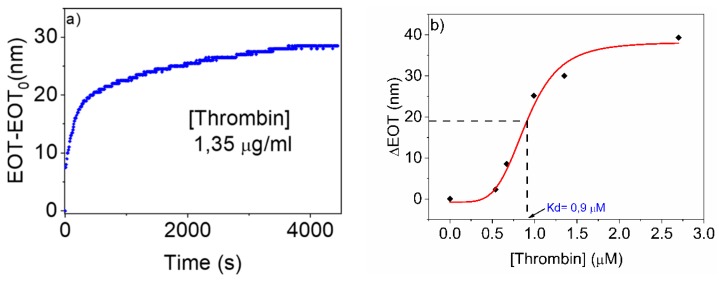
Study of thrombin detection. (**a**) Variation of EOT with time for one of the experiments performed for thrombin detection of 1.35 µM. (**b**) Absolute change in ΔEOT at different concentrations of thrombin. Red line represents a calibration curve by fitting with Boltzmann equation.

The aptamer-functionalized NAA substrates were employed to detect human thrombin protein. For this purpose, different substrates were used in RIfS experiments with human thrombin protein at different concentrations. Figure 5 shows the results of such study: Figure 5a shows one example of the evolution of the EOT signal upon infiltration of a 2.7 µM thrombin solution, while Figure 5b summarizes the response of the biosensor to the different thrombin concentrations. 

In Figure 5a, after a baseline with constant EOT corresponding to the flow of binding buffer is obtained, thrombin solution is introduced at t = 0s. At the beginning of the thrombin solution flow, a rapid increase in EOT (at a rate of 0.05 nm/s) is observed. In a second stage, the increase rate reduces, and a stable value is reached after 3618 s, with an absolute change in EOT in this step of ∆EOT = 28 nm. We can argue that in the first rapid increase mostly of the receptors are free and thrombin can rapidly bind with the aptamer, but when the receptors start to be occupied is more difficult for thrombin to access to the aptamer binding sites and the EOT ratio start to decrease.

The behaviour observed in Figure 5a is common for all the tested thrombin concentrations. With the results obtained from these experiments, the response and linearity of the TBA-functionalized NAA biosensor were studied. Figure 5b shows the absolute change in EOT as a function of different concentrations of thrombin (0, 0.54, 0.67, 0.99, 1.35 and 2.70 µM). Experiments for each concentration were conducted at least twice to ensure reproducibility. The resulting evolution of the EOT with time can be seen in the Supporting Information, Figure S3. The measured ∆EOT values show three clear regimes: very low ∆EOT (about 3 nm) at the lower concentrations, a linear increase between 0.67 µM and 1.35 µM and a saturation level at ∆EOT = 37 nm above 2 µM. This is the signature for a typical sigmoidal response curve.

The graph also shows a sigmoidal curve corresponding to the best fit to the experimental measured points of a sigmoidal function of the form:

$$\Delta EOT=A_{2}+\frac{A_{1}-A_{2}}{1+{\left(C/C_{0}\right)}^{p}}$$

where *C* is the thrombin concentration and *C*_0_, *A*_1_, *A*_2_ and the exponent p are the fitting parameters. The fitting plot (red line) shows a typical sigmoidal behaviour with an initial constant phase, a second linear phase defined as dynamic range, and finally at higher concentrations of thrombin it is produced a saturation phase. From the curve of ∆EOT versus concentration the dissociation constant K_d_ can be estimated as K_d_ = 0.9 µM.

The slope of the sigmoidal function m at its central point (C=C_0_) permits to estimate the sensitivity of the NAA sensing platform as m = 45.5 nm/µM. With this estimation of the sensitivity it is also possible to determine the limit of detection (LOD) defined as LOD = 3 × S_0_/m were S_0_ corresponds to the standard deviation of 10 single results at zero concentration and m is the slope of the linear regression curve. The estimated LOD was 7.2 nM.

### 3.3. Study of the Specificity

To investigate the specificity, the biosensing platform was exposed to a protein different than thrombin. The chosen model protein was human serum albumin (HSA) because of its globular structure similar to thrombin and because it exists in abundance in blood serum, where thrombin may also be present.

**Figure 6:**
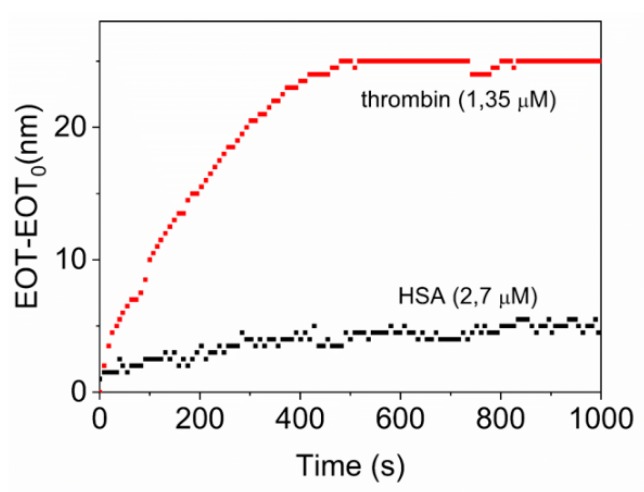
Optical response (EOT-EOT_0_) after the exposure of TBA aptamer to 1.35 µM of thrombin (**red line**) and to 2.7 µg/mL of HSA (**black line**).

Figure 6 shows the differences in the EOT variation with time whether thrombin or HSA protein are flown through the sensing system. With the flow of the 1.35 µg/mL solution of thrombin, a clear increase of the EOT is observed until it reaches a stable value corresponding to a ∆EOT = 25 nm. Instead, the flow of a more concentrated HSA solution (2.7 µg/mL) does not produce a remarkable change in EOT, and only a change of ∆EOT = 4 nm is observed.

**Figure 7:**
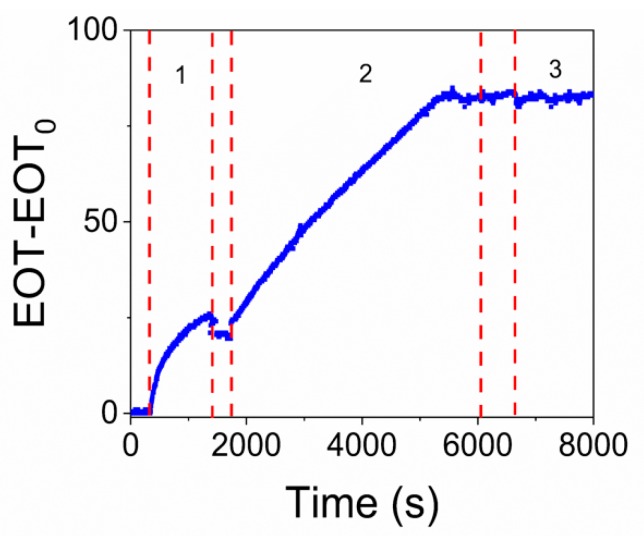
EOT evolution during the sulfo-NHS-biotin (**1**), streptavidin (**2**) and thrombin infiltration (**3**) without TBA.

Finally, to determine the proper TBA immobilization and the non-specific binding of the thrombin directly to the NAA surface or to the other earlier immobilized molecules (e.g., biotin and streptavidin), a further experiment consisting of the flow of thrombin just after stage 2 of the functionalization process (streptavidin attachment) was performed. Figure 7 shows the evolution of EOT during sulfo-NHS-biotin grafting in stage 1 of the functionalization process, and streptavidin attachment in stage 2. Finally, in the step labelled as 3 in the figure, a solution of 2.7 µM of thrombin was introduced. The exposition of biosensor platform to thrombin without addition of TBA did not produce any noticeable change in EOT.

## 4. Conclusions

In this work we demonstrate the ability of nanoporous anodic alumina-based platforms to be used in aptabiosensors with real-time detection of the different steps of the functionalization and biosensing process. First, the optimal functionalization pathway to immobilize thrombin binding aptamer has been found and demonstrated by real-time monitoring by means of RIfS. A high reproducibility of the three stages in the functionalization has been demonstrated.

The functionalized substrates are subsequently used to prove the ability of the NAA-RIfS system to detect thrombin in the µM range whit a limit of detection of 7.2 nM and a sensitivity of 45.5 nm/µM. Furthermore, the high specificity of the system to detect thrombin without cross-reaction with another very similar molecule has been proven.

This study opens the possibility to use NAA in flow RIfS system as a promising platform for new very specific and sensitive biosensors. This system provides the possibility to develop new analytic devices based on aptamers or other biotinylated receptors (e.g., antibodies, enzymes, proteins…), to detect several types of targets (e.g., proteins, cells or even very small molecules).

## Supplementary Materials

The following are available online at https://www.mdpi.com/1424-8220/19/20/4543/s1, Figure S1: SEM pictures of one of the NAA samples prepared as basis for the biosensing platform, Figure S2: variation of EOT for the functionalization steps in triplicate experiments, Figure S3: variation of EOT for the Thrombin detection step for the different studied concentrations, in duplicate experiments.
