# Supplementary material for: Aptamer-Based Nanoporous Anodic Alumina Interferometric Biosensor for Real-Time Thrombin Detection
Source: Sensors (Basel). 2019 Oct 19;19(20):4543. doi: 10.3390/s19204543 (PMC6833485; doi:10.3390/s19204543)
Supplement: Supplementary file 1 [file sensors-19-04543-s001.pdf]

SUPPLEMENTARY MATERIAL

Aptamer-based Nanoporous Anodic Alumina Interferometric Biosensor for Real-time Thrombin Detection

Laura Pol, Laura Karen Acosta, Josep Ferré-Borrull and Lluis F. Marsal*

*Corresponding author: lluis.marsal@urv.cat

Universitat Rovira i Virgili

Group of Nano-electronic and Photonic Systems (NePhoS)

Departament d'Enginyeria Electrònica, Elèctrica i Automàtica, Avda. Països Catalans 26, 43007 Tarragona, Spain

Figure S1: ESEM image of NAA 60 nm of pore diameter a) top and b) cross section

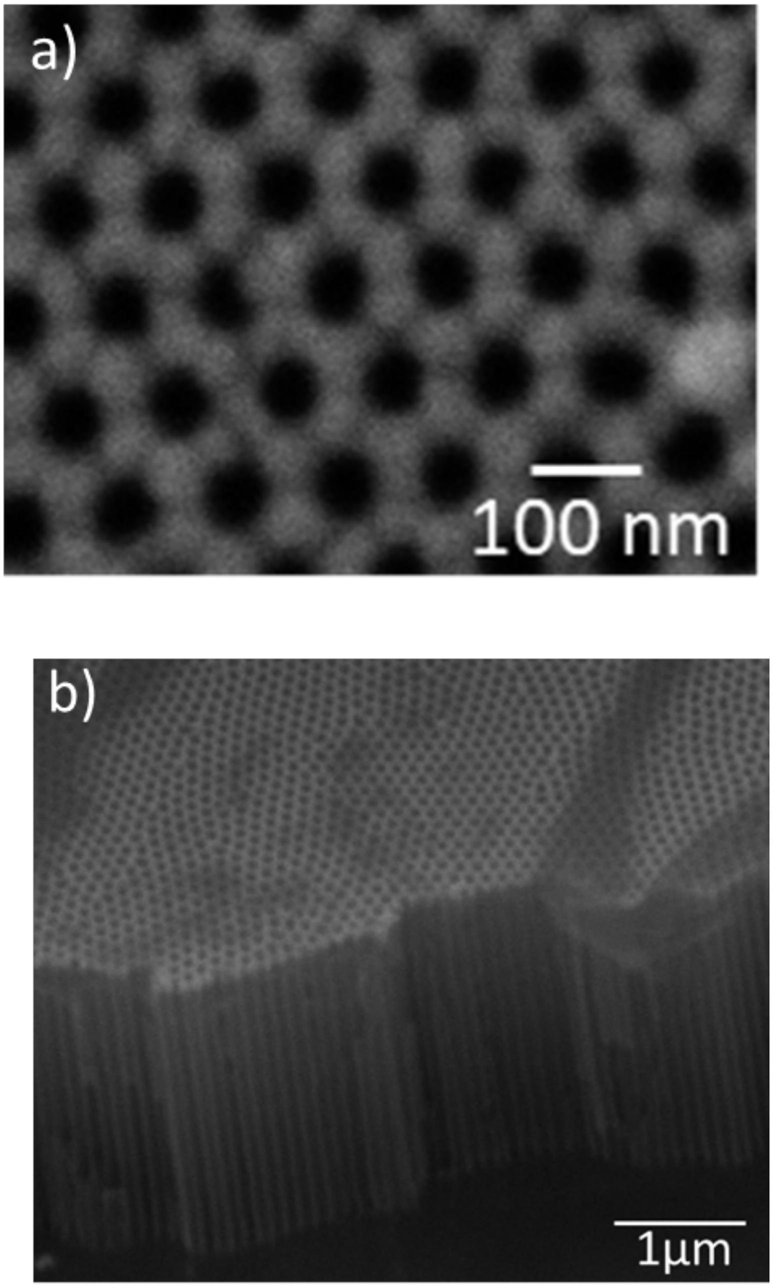

Figure S2: Registered change in EOT as a function of time for complete biotinylated TBA attachment experiments before the detection of different concentrations of thrombin. The different steps in the experiment are indicated in the graphs as numbers and correspond to: 1=Sulfo-NHS-Biotin, 2 = Streptavidin, 3 = Biotinylated TBA.

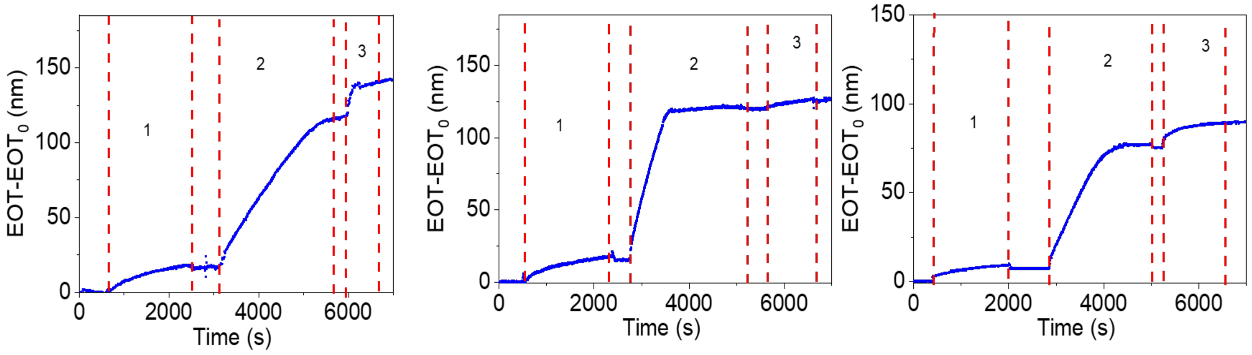

Figure S3: Registered change in EOT as a function of time for two replicas at different concentrations of thrombin a) and b) 2.7 µM, c and d) 1.35 µM, e) and f) 0.99, g) and h) 0.67 µM, i) and j) 0,54 µM

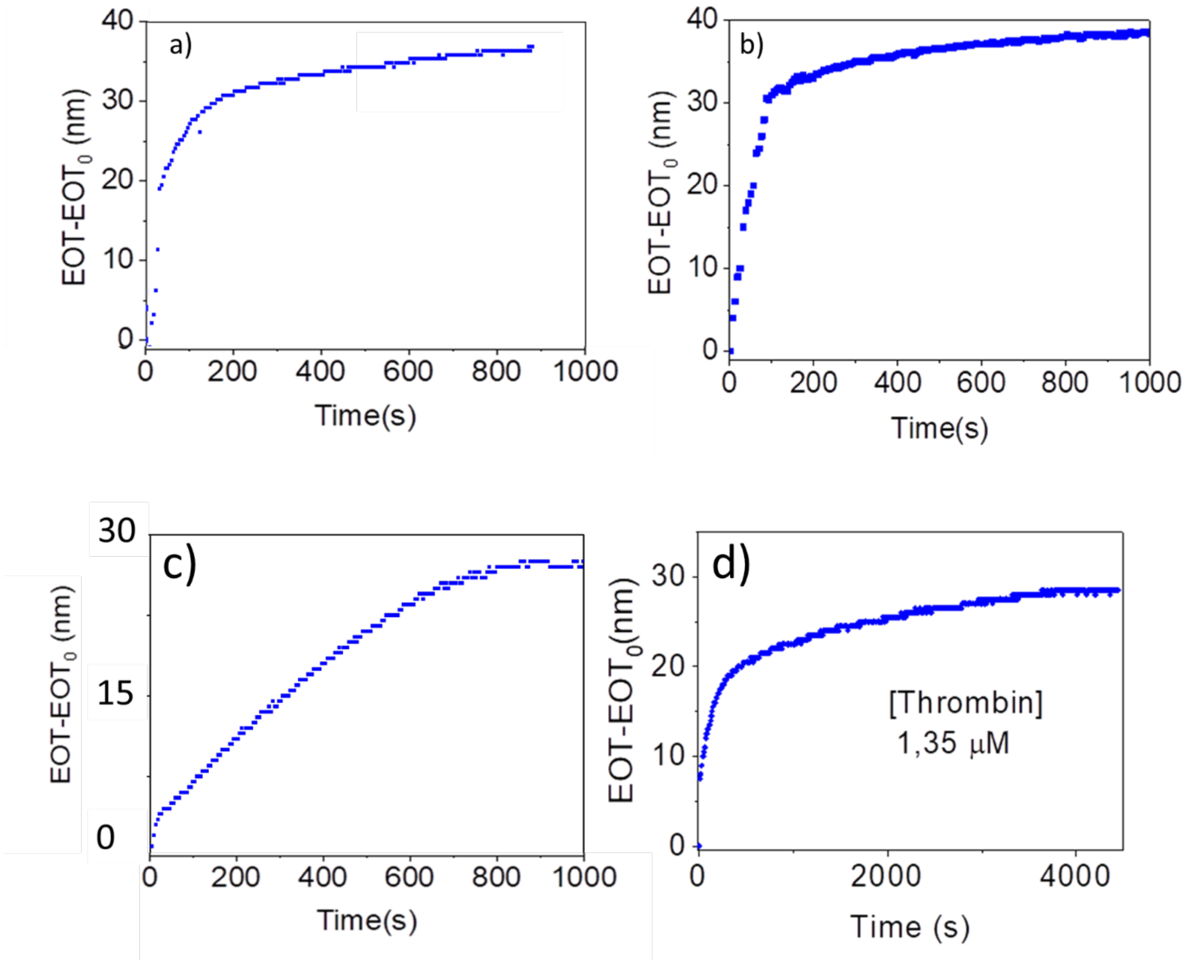

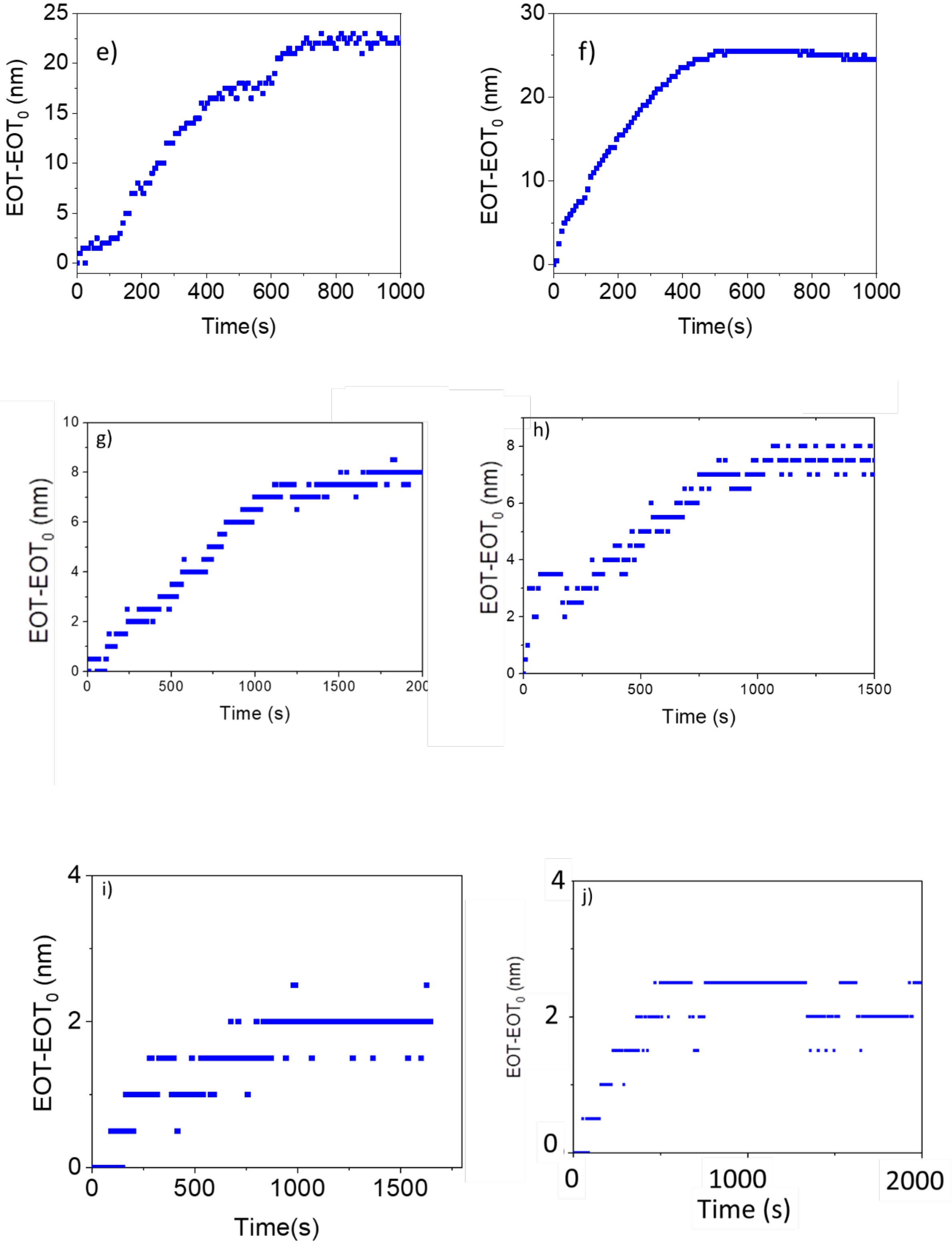

e)
EOT-EOT0 (nm)
0
5
10
15
20
25
0
200
400
600
800
1000
Time(s)
f)
EOT-EOT0 (nm)
0
10
20
30
0
200
400
600
800
1000
Time(s)
g)
EOT-EOT0 (nm)
0
2
4
6
8
10
0
500
1000
1500
200
Time (s)
h)
EOT-EOT0 (nm)
0
2
4
6
8
0
500
1000
1500
Time (s)
i)
EOT-EOT0 (nm)
0
2
4
0
500
1000
1500
Time(s)
j)
EOT-EOT0 (nm)
0
2
4
0
1000
2000
Time (s)
